# Explainable AI: A review of applications to neuroimaging data

**DOI:** 10.3389/fnins.2022.906290

**Published:** 2022-12-01

**Authors:** Farzad V. Farahani, Krzysztof Fiok, Behshad Lahijanian, Waldemar Karwowski, Pamela K. Douglas

**Affiliations:** ^1^Department of Biostatistics, Johns Hopkins University, Baltimore, MD, United States; ^2^Department of Industrial Engineering and Management Systems, University of Central Florida, Orlando, FL, United States; ^3^Department of Industrial and Systems Engineering, University of Florida, Gainesville, FL, United States; ^4^H. Milton Stewart School of Industrial and Systems Engineering, Georgia Institute of Technology, Atlanta, GA, United States; ^5^School of Modeling, Simulation, and Training, University of Central Florida, Orlando, FL, United States

**Keywords:** explainable AI, interpretability, artificial intelligence (AI), deep learning, neural networks, medical imaging, neuroimaging

## Abstract

Deep neural networks (DNNs) have transformed the field of computer vision and currently constitute some of the best models for representations learned *via* hierarchical processing in the human brain. In medical imaging, these models have shown human-level performance and even higher in the early diagnosis of a wide range of diseases. However, the goal is often not only to accurately predict group membership or diagnose but also to provide explanations that support the model decision in a context that a human can readily interpret. The limited transparency has hindered the adoption of DNN algorithms across many domains. Numerous explainable artificial intelligence (XAI) techniques have been developed to peer inside the “black box” and make sense of DNN models, taking somewhat divergent approaches. Here, we suggest that these methods may be considered in light of the interpretation goal, including functional or mechanistic interpretations, developing archetypal class instances, or assessing the relevance of certain features or mappings on a trained model in a *post-hoc* capacity. We then focus on reviewing recent applications of *post-hoc* relevance techniques as applied to neuroimaging data. Moreover, this article suggests a method for comparing the reliability of XAI methods, especially in deep neural networks, along with their advantages and pitfalls.

## Introduction

Machine learning (ML) and deep learning (DL; also known as hierarchical learning or deep structured learning) models have revolutionized computational analysis (Bengio et al., 2013; LeCun et al., 2015; Schmidhuber, 2015) across a variety of fields such as text parsing, facial reconstruction, recommender systems, and self-driving cars (Cheng et al., 2016; Richardson et al., 2017; Young et al., 2018; Grigorescu et al., 2020). These models are particularly successful when applied to images, achieving human-level performance on visual recognition tasks (Kriegeskorte, 2015; LeCun et al., 2015). Among the demanding domains confronting ML/DL researchers are healthcare and medicine (Litjens et al., 2017; Miotto et al., 2017; Shen et al., 2017; Kermany et al., 2018). In medical imaging and neuroimaging, in particular, deep learning has been used to make new discoveries in various domains. For example, conventional wisdom for radiologists was that little or no prognostic information is contained within a tumor, and therefore one should examine its borders. However, a recent deep learning approach coupled to texture analysis found predictive information within the tumor itself (Alex et al., 2017). As another example, Esteva et al. (2017) demonstrated that a single convolutional neural network could classify skin cancer with high predictive performance, on par with the performance of a dermatologist. Numerous other studies have been conducted on various intelligent medical imaging fields, from diabetic retinopathy (Ting et al., 2017) up to lung cancer (Farahani et al., 2018) and Alzheimer's disease (Tang et al., 2019), all of which demonstrate good predictive performance.

However, in practice, data artifacts might compromise the high performance of ML/DL models, making it difficult to find a suitable problem representation (Leek et al., 2010). Ideally, though, these algorithms could be leveraged for both prediction and explanation, where the latter may drive human discovery of improved ways to solve problems (Silver et al., 2016; Hölldobler et al., 2017). Thus, strategies for comprehending and explaining what the model has learned are crucial to deliver a robust validation scheme (Došilović et al., 2018; Lipton, 2018; Montavon et al., 2018), particularly in medicine (Caruana et al., 2015) and neuroscience (Sturm et al., 2016), which must be modeled based on correct features. For example, brain tumor resection requires an interpretation in a feature space that humans can readily understand, such as image or text, to leverage that information in an actionable capacity (Mirchi et al., 2020; Pfeifer et al., 2021).

Conventionally, to compare a new ML/DL technique to the existing gold standard in medicine (i.e., the human in most applications), the sensitivity, specificity, and predictive values are first calculated for each modality. Then, the confusion matrices could be constructed for both the new technique and the clinician (e.g., radiologist) and ultimately compared with each other. However, a significant weakness of this comparison is that it ignores the similarity between the support features of the ML/DL model (e.g., voxels, pixels, edges, etc.) and the features examined by the radiologist (e.g., hand-drawn or eye-tracking features). Accordingly, it is impossible to determine whether the model has learned from the embedded signals or from the artifacts or didactic noise that covary with the target (Goodfellow I. J. et al., 2014; Montavon et al., 2017; Douglas and Farahani, 2020). In other words, the presence of adversarial noise, which could be simply introduced due to instrumentation, prevents achieving a robust explanation of model decisions.

It is widely accepted that the different architectures of DL methods, e.g., recurrent neural network (RNN), long short term memory (LSTM), deep belief network (DBN), convolutional neural network (CNN), and generative adversarial network (GAN), which are well-known for their high predictive performance, are effectively considered to be black boxes, with internal inference engines that users cannot interpret (Guidotti et al., 2018b). Therefore, the limited transparency and explainability in such non-linear methods has prevented their adoption throughout the sciences; as a result, simpler models with higher interpretability (e.g., shallow decision trees, linear regression, or non-negative matrix factorization) remain more popular than complex models in many applications, including bioinformatics and neuroscience though these choices often reduce predictivity (Ma et al., 2007; Devarajan, 2008; Allen et al., 2012; Haufe et al., 2014; Bologna and Hayashi, 2017). Traditionally, some researchers believe that there is a trade-off between prediction performance and explainability for commonly used ML/DL models (Gunning and Aha, 2019). In this respect, decision trees presumably exhibit the highest explainability but are the least likely to deliver accurate results, whereas DL methods represent the best predictive performance and the worst model explainability. However, it is essential to underline that this notion has no proven linear relationship, and it can be bent for specific models/methods and sophisticated setups (Yeom et al., 2021), increasing both prediction performance and explainability.

Recently, this notion has been strongly challenged by novel explainable AI studies, in which well-designed interpretation techniques have shed light on many deep non-linear machine learning models (Simonyan et al., 2013; Zeiler and Fergus, 2014; Bach et al., 2015; Nguyen et al., 2016; Ribeiro et al., 2016; Selvaraju et al., 2017; Montavon et al., 2018; Hall and Gill, 2019; Holzinger et al., 2019). Remarkably, in healthcare and medicine, there is a growing demand for building AI approaches that must perform well and guarantee transparency and interpretability to medical experts (Douglas et al., 2011; Holzinger et al., 2019). Additionally, researchers suggest keeping humans in the loop—considering expert knowledge in interpreting the ML/DL results—leads to user trust and identifying points of model failure (Holzinger, 2016; Magister et al., 2021). In recognition of the importance of transparency in models defined for the medical imaging data, dedicated datasets and XAI exploration environments were recently proposed (Holzinger et al., 2021). Due to the nascent nature of the neuroimaging filed and its extensive use in deep learning studies, techniques such as magnetic resonance imaging (MRI), functional MRI (fMRI), computerized tomography (CT), and ultrasound, have considerably piqued the interest of XAI researchers (Zhu et al., 2019; van der Velden et al., 2022).

The present work provides a systematic review of recent neuroimaging studies that have introduced, discussed, or applied the *post-hoc* explainable AI methods. The *post-hoc* methods take a fitted and trained model and extract information about the relationships between the model input and model decision (with no effect on model performance). In contrast, model-based approaches alter the model to allow for mechanistic (functional) or archetypal explanations. In this work, we focused on *post-hoc* methods of their importance for practitioners and researchers who deal with deep neural networks and neuroimaging techniques. Hence, standard data analysis methods can be utilized to evaluate the extracted information and provide tangible outcomes to the end users. The remaining sections are organized as follows. Section Background: Approaches for interpreting DNNs summarizes the existing techniques for interpreting deep neural networks, categorized into saliency (e.g., gradients, signal, and decomposition) and perturbation methods. Section Methodology discusses our search strategy for identifying relevant publications and their inclusion criterion and validity risk assessment. Section Results provides the results of a literature search, study characteristics, reliability analysis of XAI methods, and quality assessment of the included studies. Finally, section Discussion discusses the significant limitations of XAI-based techniques in medical domains and highlights several challenging issues and future perspectives in this emerging field of research.

## Background: Approaches for interpreting DNNs

**Figure 1:**
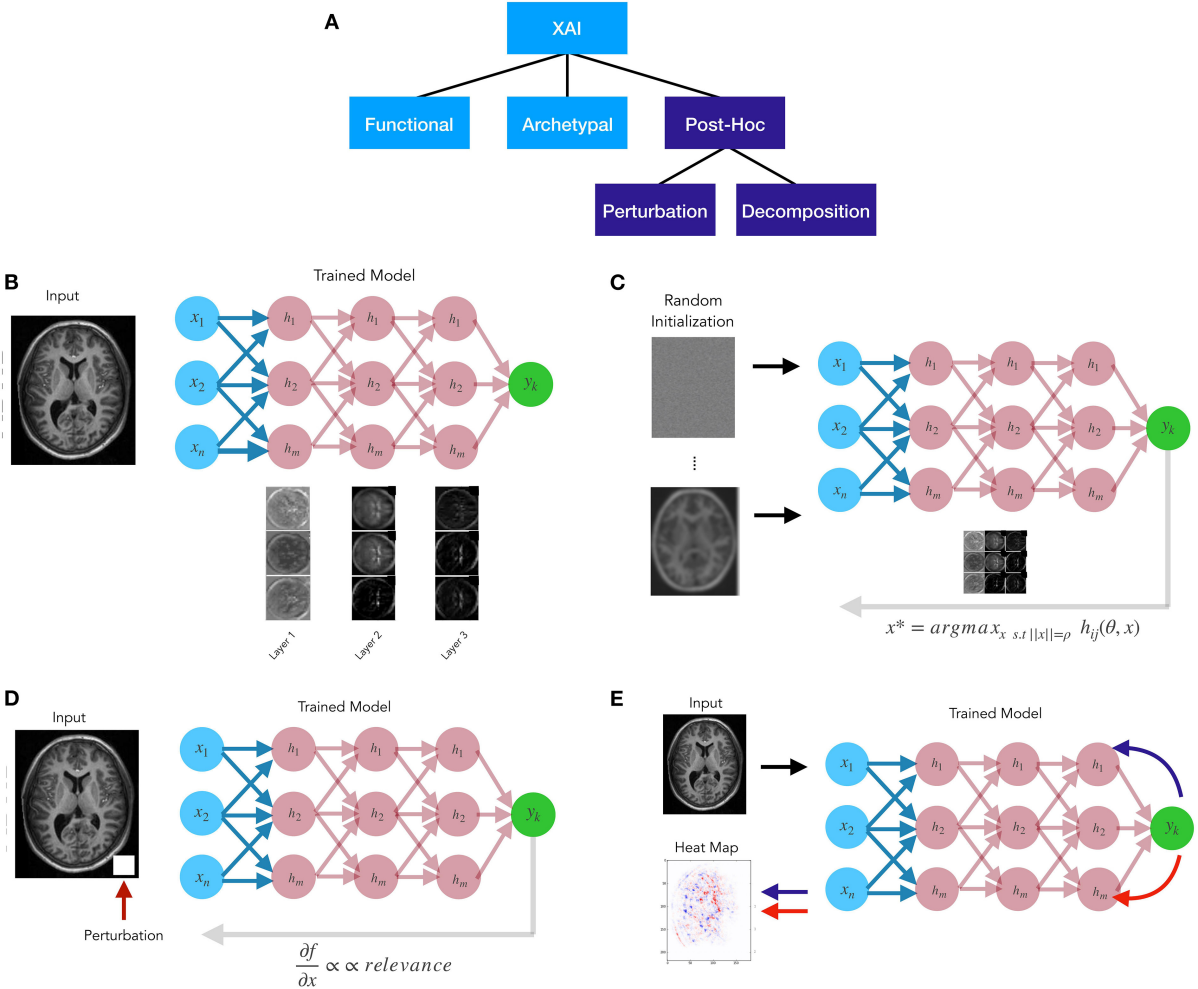
**(A)** Explainable AI methods taxonomy. **(B)** Functional approaches attempt to disclose the algorithm's mechanistic aspects. **(C)** Archetypal approaches, like generative methods, seek to uncover input patterns that yield the best model response. **(D)** *Post-hoc* perturbation relevance approaches generally change the inputs or the model's components and then attributing relevance proportionally to the amount of the change in model output. **(E)** *Post-hoc* decomposition relevance approaches are propagation-based techniques explaining an algorithm's decisions by redistributing the function value (i.e., the neural network's output) to the input variables, often in a layer-by-layer fashion.

A variety of explainable AI (XAI) techniques have been developed in recent years that have taken various approaches. For example, some XAI methods are model agnostic, and some take a local as opposed to a global approach. Some have rendered heatmaps based on “digital staining” or combining weights from feature maps in the last hidden layer (Cruz-Roa et al., 2013; Xu et al., 2017; Hägele et al., 2020). Here we suggest that these methods should be distinguished based on the goal of the explanation: functional, archetypal, or *post-hoc* (relevance) approximation (Figure 1A).

*Functional* approaches (Figure 1B) examine the learned representations in the graph to reveal mechanistic aspects of the algorithm (Khaligh-Razavi and Kriegeskorte, 2014; Kriegeskorte and Douglas, 2018). One of the goals of these approaches is to shed light onto how the feature maps or filters learned through the layers help the model achieve its goal, or support its global decision structure. *Archetypal* methods (Figure 1C) attempt to find an input pattern *x* that is a prototypical exemplar of *y*. A simple example of this is activation maximization, whereby an initial input is randomized and the algorithm searches for input patterns that produce maximal response from the model (Erhan et al., 2009). A variety of generative methods have been developed for archetypal purposes, such as generative adversarial network models (Goodfellow I. et al., 2014). *Post-hoc* (or *relevance*) methods (Figures 1D,E) attempt to determine which aspects of input *x* make it likely to take on a group membership or provide supporting evidence for a particular class. In general relevance methods fall into three classes: feature ranking, perturbation methods, and decomposition methods. Feature ranking as well as feature selection methods have existed for many years (e.g., Guyon and Elisseeff, 2003), though their importance or lack thereof in high dimensional medical imaging data sets has been debated (Chu et al., 2012; Kerr et al., 2014). *Perturbation* relevance methods (Figure 1D) provide a local estimate of the importance of an image region or feature. This class of techniques involve altering some aspect of the inputs or the model, and subsequently assigning relevance as proportional to the magnitude of the alteration in the model output. An initial example of this is the classic pixel flip (Bach et al., 2015; Samek et al., 2017a), whereby small local regions of the image are altered, and the ensuing changes to the output are mapped back in the form of a relevance score to those altered pixels. Alternatively, perturbation methods may alter the weights, and see how this effects the output *f(x)*. Perturbations methods can be model agnostic or not, depending upon their implementation. Lastly, *decomposition* or *redistribution* methods (Figure 1E) for relevance assignment attempt to determine the share of relevance through the model layers by examining the model structure, and are thus, typically model dependent. These attribute methods involve message passing, and propose relevance backward through the model, viewing prediction as the output of a computational graph.

### History of *post-hoc* explanation techniques

The earliest work in XAI can be traced to 1960–1980, when some expert systems were equipped with rules that could interpret their results (McCarthy, 1960; Shortliffe and Buchanan, 1975; Scott et al., 1977). Although the logical inference of such systems was easily readable by humans, many of these systems were never used in practice for poor predictive performance, lack of generalizability, and the high cost of their knowledge base maintenance (Holzinger et al., 2019). The emergence of ML techniques, especially those based on deep neural networks, has overcome many traditional limitations, although their interpretability to users remains their primary challenge (Lake et al., 2017).

Accordingly, in recent years, AI researchers have afforded considerable focus to peering inside the black-box of DNNs and enhancing the system's transparency (Baehrens et al., 2009; Anderson et al., 2012; Haufe et al., 2014; Simonyan and Zisserman, 2014; Springenberg et al., 2014; Zeiler and Fergus, 2014; Bach et al., 2015; Yosinski et al., 2015; Nguyen et al., 2016; Kindermans et al., 2017; Montavon et al., 2017; Smilkov et al., 2017; Sundararajan et al., 2017; Zintgraf et al., 2017). In the following, we review the latest methods used to interpret deep learning models, including perturbation and decomposition (or redistribution) approaches. While unified in purpose, i.e., revealing the relationship between inputs and outputs (or higher levels) of the underlying model, these methods are highly divergent in outcome and explanation mechanism. The *post-hoc* XAI methods we found in our review are listed in Table 1 and discussed in the following subsections.

**Table 1:** *Post-hoc* methods for interpreting deep neural networks.

**Taxonomy**		**Method**	**References**
Perturbation	Gradients (sensitivity)	N/A (gradient-based)	Baehrens et al., 2009
		Saliency maps	Simonyan et al., 2013
		Class activation mapping (CAM)	Zhou et al., 2016
		Gradient-weighted CAM (Grad-CAM)	Selvaraju et al., 2017
		Guided Grad-CAM	Selvaraju et al., 2016
		3D CAM	Yang et al., 2018
		3D Grad-CAM	Yang et al., 2018
		Respond-CAM	Zhao et al., 2018
		Multiscale CAM	Hu et al., 2020
		SmoothGrad (SG)	Smilkov et al., 2017
		Correlation maps	Schirrmeister et al., 2017
		Testing with concept activation vectors (TCAV)	Kim et al., 2018
		Automated concept-based explanation (ACE)	Ghorbani et al., 2019a,b
	Signal	Guided backpropagation (GBP)	Springenberg et al., 2014
		DeConvNet (occlusion maps)	Zeiler and Fergus, 2014
		Inversion-based	Mahendran and Vedaldi, 2015
		Inversion-based	Dosovitskiy and Brox, 2016
		PatternNet	Kindermans et al., 2017
		PatternAttribution	Kindermans et al., 2017
	Model agnostic	Local interpretable model-agnostic explanations (LIME)	Ribeiro et al., 2016
		Submodular pick LIME (SP-LIME)	Ribeiro et al., 2016
		anchor-LIME (aLIME)	Tulio Ribeiro et al., 2016
		Model agnostic globally interpretable explanations	Puri et al., 2017
		SHapley additive exPlanations (SHAP)	Lundberg and Lee, 2017
Decomposition (redistribution)		Layer-wise relevance propagation (LRP)	Bach et al., 2015
		Deep Taylor decomposition	Montavon et al., 2017
		Deep learning important FeaTures (DeepLIFT)	Shrikumar et al., 2017
		Integrated gradients (IG)	Sundararajan et al., 2017
		Gradient × input	Shrikumar et al., 2017
		Prediction difference analysis (PDA)	Zintgraf et al., 2017
		Graph LRP	Chereda et al., 2021

#### Perturbation approach

The perturbation-based approach is broadly divided into model-specific (e.g., gradients and signal) or model-agnostic methods. Gradients/sensitivity-based methods examine how a slight shift to the input affects the classification score for the output of interest, such as the techniques introduced by Baehrens et al. (2009) and Simonyan et al. (2013), as well as Class Activation Mapping (CAM; Zhou et al., 2016), Gradient-weighted CAM (Grad-CAM; Selvaraju et al., 2017), SmoothGrad (SG; Smilkov et al., 2017), and (Multiscale CAM; Hu et al., 2020). These techniques are easily implemented in DNNs because the gradient is generally computed by backpropagation (Rumelhart et al., 1986; Swartout et al., 1991). Signal methods typically visualize input patterns by stimulating neuron activation in higher layers, resulting in so-called feature maps. DeConvNet (Zeiler and Fergus, 2014), Guided BackProp (Springenberg et al., 2014), PatternNet (Kindermans et al., 2017), and inversion-based techniques (Mahendran and Vedaldi, 2015; Dosovitskiy and Brox, 2016) are some examples of this group. Mahendran and Vedaldi (2015) showed that by moving from the shallower layers to the deeper layers, the feature maps reveal more complex patterns of input [e.g., in human face explanation: from (1) line and edges to (2) eyes, nose, and ears, then to (3) complex facial structures].

On the other hand, model agnostic methods explore the prediction of interest to infer the relevance of the input features toward the output (Ribeiro et al., 2016; Alvarez-Melis and Jaakkola, 2018). Two of the most popular techniques in this category are Local Interpretable Model-agnostic Explanations (LIME; Ribeiro et al., 2016) and SHapley Additive exPlanations (SHAP; Lundberg and Lee, 2017).

#### Decomposition approach

Decomposition-based methods seek to identify important features (pixels) in a particular input by decomposing the network classification decision into contributions of the input elements. The earliest study in this class goes back to Bach et al. (2015), who introduced the Layer-Wise Relevance Propagation (LRP) technique, which interprets the DNN decisions using heatmaps (or relevance-maps). Using a set of propagation rules, LRP performs a separate backward pass for each possible target class, satisfying a layer-wise conservation principle (Landecker et al., 2013; Bach et al., 2015). As a result, each intermediate layer up to the input layer is assigned relevance scores. The sum of the scores in each layer equals the prediction output for the class under consideration. The conservation principle is one of the significant differences between the decomposition and gradients methods.

In another technique, Montavon et al. (2017) demonstrated how the propagation rules derived from deep Taylor decomposition relate to those heuristically defined by Bach et al. (2015). Recently, several studies have used LRP to interpret and visualize their network decisions in various applications such as text analysis (Arras et al., 2017a), speech recognition (Becker et al., 2018), action recognition (Srinivasan et al., 2017), and neuroimaging (Thomas et al., 2019). Other recently-developed decomposition-based methods include DeepLIFT (Shrikumar et al., 2017), Integrated Gradients (Sundararajan et al., 2017), Gradient Input (Shrikumar et al., 2017), and Prediction Difference Analysis (PDA; Zintgraf et al., 2017). In recent years, various studies have attempted to test the reliability of explanation techniques compared to each other by introducing several properties such as fidelity (or sensitivity), consistency, stability and completeness (Bach et al., 2015; Kindermans et al., 2017; Sundararajan et al., 2017; Alvarez-Melis and Jaakkola, 2018). In the following chapters, we address this issue.

## Methodology

This systematic review was conducted according to the PRISMA (Preferred Reporting Items for Systematic Reviews and Meta-Analyses) statement guidelines (Moher et al., 2009). To reduce the effect of research expectations on the review, we first identified research questions and search strategies. Moreover, this systematic review adhered to the Cochrane Collaboration methodology (Higgins et al., 2011), to mitigate the risk of bias and error.

Based on the objectives outlined in the abstract, the following research questions were derived and form the cornerstone of our study:

- What are the main challenges in AI that have limited their implementation in medical imaging applications, particularly in neuroimaging, despite their high prediction performance?
- How can we overcome the black-box property of complex and deep neural networks for the user in critical areas such as healthcare and medicine?
- How have recent advances in explainable AI affected machine/deep learning in medical imaging and neuroimaging?
- How can one assess the reliability and generalizability of interpretation techniques?

### Search strategy

The current and seminal studies in the realm of XAI with a focus on healthcare and medicine were considered critical sources for this systematic review. A bibliographic search for this work was carried out across the following scientific databases and search engines: PubMed, Scopus, Web of Science, Google Scholar, ScienceDirect, IEEE Xplore, SpringerLink, and arXiv, using the following keyword combinations in the title, keywords, or abstract: (“explainable AI” or “XAI” or “explainability” or “interpretability”) and (“artificial intelligence” or “machine learning” or “deep learning” or “deep neural networks”) and (“medical imaging” or “neuroimaging” or “MRI” or “fMRI” or “CT”). Moreover, the reference lists of the retrieved studies were also screened to find relevant published works.

### Inclusion criteria

Published original articles with the following features were included in the current study: (a) be written in English; AND [(b) introduce, identify, or describe XAI-based techniques for visualizing and/or interpreting ML/DL decisions; OR (c) be related to the application of XAI in healthcare and medicine]. Other exclusion criteria were: (a) book chapters; (b) papers that upon review were not related to the research questions; (c) opinions, viewpoints, anecdotes, letters, and editorials. The eligibility criteria were independently assessed by two authors (FF and KF), who screened the titles and abstracts to establish the relevant articles based on the selection criteria. Any discrepancies were resolved through discussion or referral to a third reviewer (BL or WK).

### Data extraction

We developed a data extraction sheet, pilot-tested this sheet on randomly selected studies, and refined the sheet appropriately. During a full-text review process, one review author (KF) extracted the following data from the selected studies, and a second review author (FF) crosschecked the collected data, which included: taxonomic topic, first author (year of publication), key contributions, XAI model used, and sample size (if applicable). Disagreements were resolved by discussion between the two review authors, and if necessary, a third reviewer was invoked (BL or WK).

### Additional analyses

We performed a co-occurrence analysis to analyze text relationships between the shared components of the reviewed studies, including XAI methods, imaging modalities, diseases, and frequently used ML/DL terms. Creating a co-occurrence network entails finding keywords in the text, computing the frequency of co-occurrences, and analyzing the networks to identify word clusters and locate central terms (Segev, 2020). Furthermore, to provide a critical view of the extracted XAI techniques, we carried out an additional subjective examination of articles that have proposed quality tests for evaluating the reliability of these methods.

### Quality assessment

The risk of bias in individual studies was ascertained independently by two reviewers (FF and KF) following the Cochrane Collaboration's tool (Higgins et al., 2011). The Cochrane Collaboration's tool assesses random sequence generation, allocation concealment, blinding of participants, blinding of outcome assessment, incomplete outcome data, and selective outcome reporting, and ultimately rates the overall quality of the studies as weak, fair, or good. To appraise the quality of evidence across studies, we examined for lack of completeness (publication bias) and missing data from the included studies (selective reporting within studies). The risk of missing studies is highly dependent on the chosen keywords and the limitations of the search engines. A set of highly-cited articles was used to create the keyword search list in an iterative process to alleviate this risk. Disagreements were resolved by discussion between the study authors.

## Results

### Literature search

**Figure 2:**
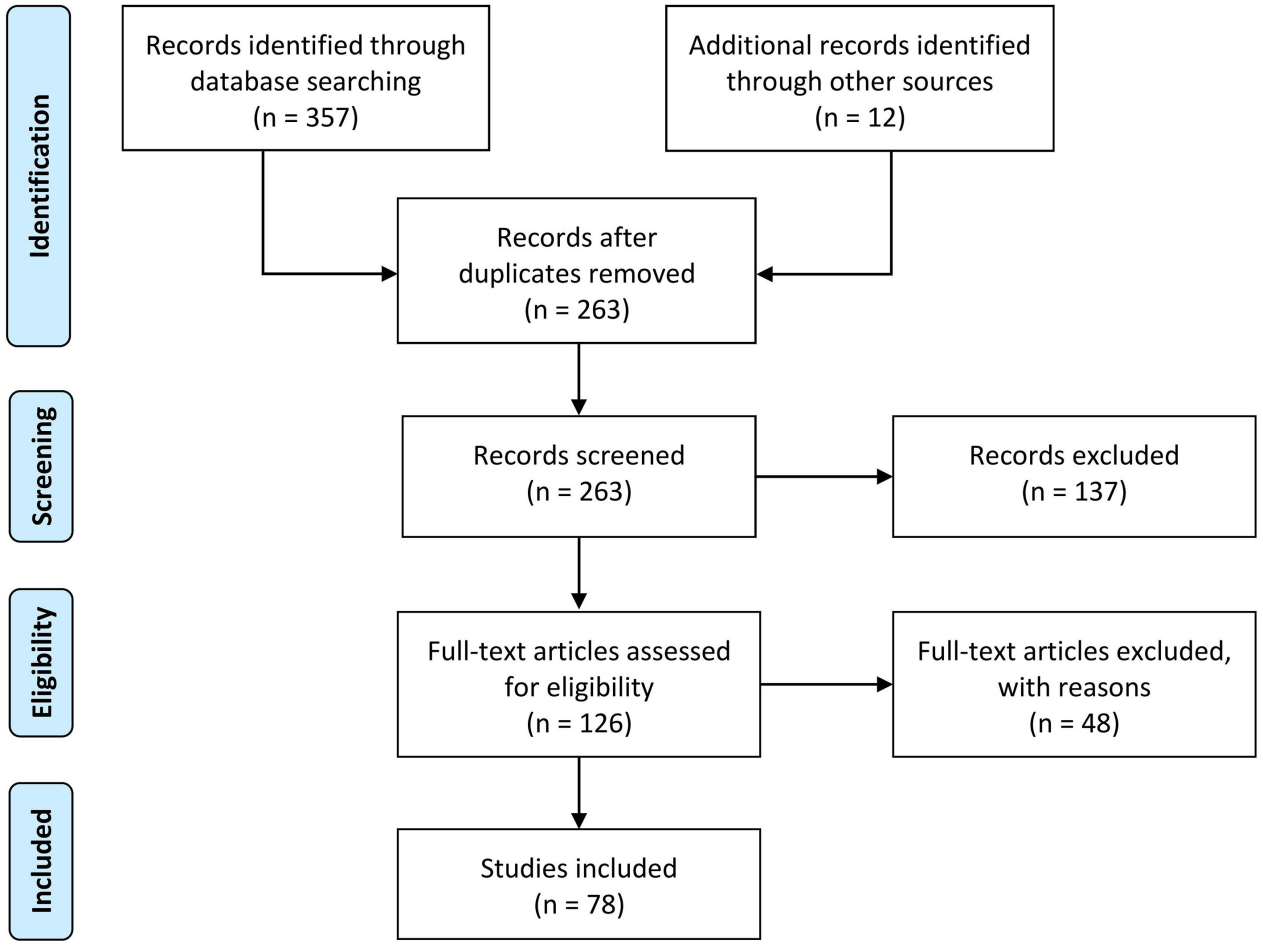
The flow diagram of the methodology and selection processes used in this systematic review follows the PRISMA statement (Moher et al., 2009).

Following the PRISMA guidelines (Moher et al., 2009), a summary of the process used to identify, screen, and select studies for inclusion in this review is illustrated in Figure 2. First, 357 papers were identified through the initial search, followed by the removal of duplicate articles, which resulted in 263 unique articles. Only ~5% were published before 2010, indicating the novelty of the terminology and the research area. Afterwards, the more relevant studies were identified from the remaining papers by incorporating inclusion and exclusion criteria. The inclusion criteria at this step required the research to (a) be written in English; AND [(b) introduce, identify, or describe *post-hoc* XAI techniques for visualizing and/or interpreting ML/DL decisions; OR (c) be related to the application of *post-hoc* XAI in neuroimaging]. Other exclusion criteria included: (a) book chapters; (b) papers which upon review were not related to the research questions; (c) opinions, viewpoints, anecdotes, letters, and editorials. As a result, the implementation of these criteria yielded 126 eligible studies (~48% of the original articles). Subsequently, the full text of these 126 papers was scrutinized in detail to reaffirm the criteria described in the previous step. Eventually, 78 publications remained for systematic review.

### Study characteristics

**Figure 3:**
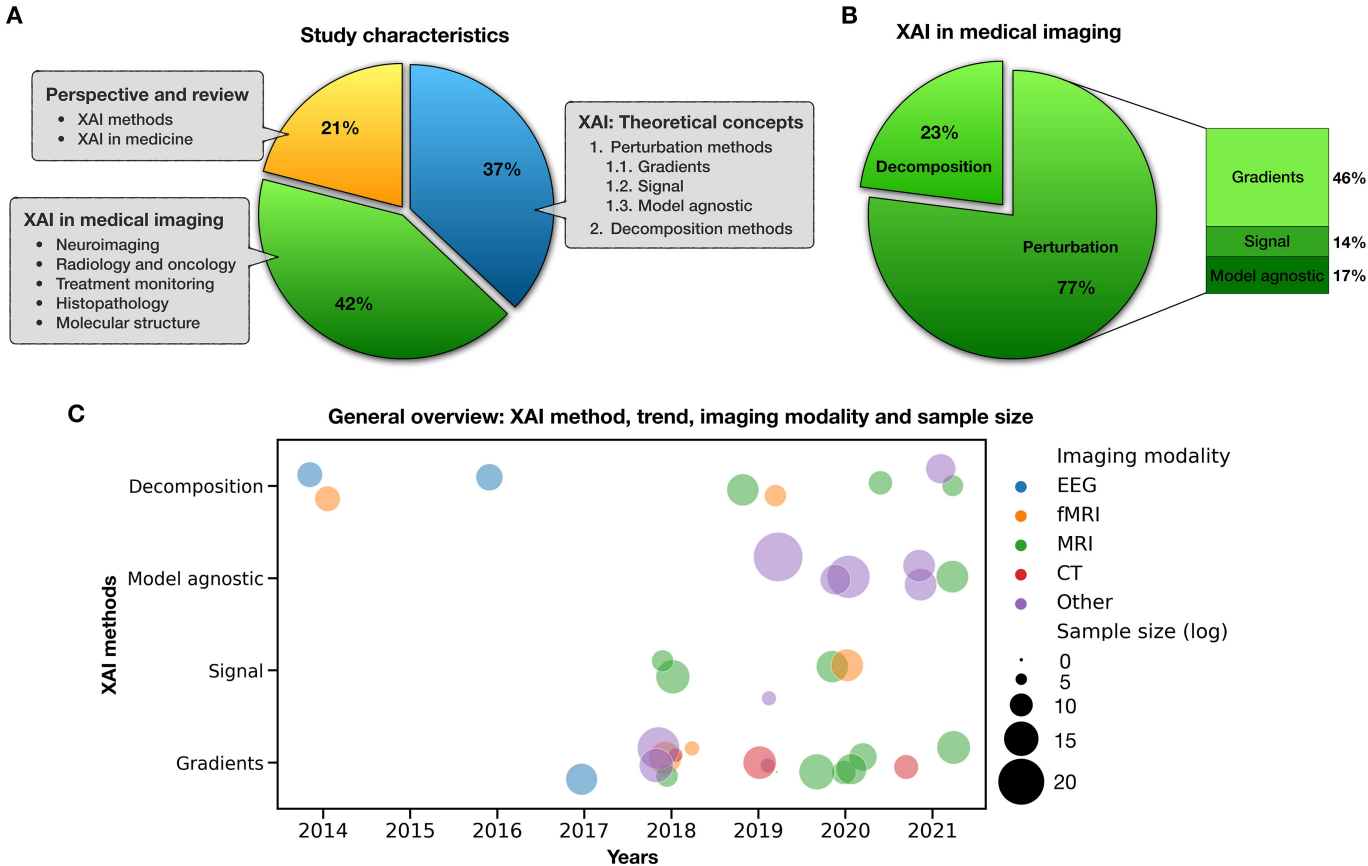
Study characteristics. **(A)** Categorization of included studies, **(B)** XAI in medical imaging, and **(C)** a bubble plot that shows mentioned studies by type of XAI method, imaging modality, sample size, and publication trend in recent years.

The included studies—published from 2005 to 2021—were binned into three major taxonomies (Figure 3A). The first category was focused on the researchers' efforts to introduce *post-hoc* XAI techniques and theoretical concepts for the visualization and interpretation of deep neural network predictions (blue slice) and accounted for 37% of the selected articles. In the second category, articles discussing the neuroimaging applications of XAI were collected and reviewed (green slice), accounting for 42% of the selected papers. Finally, the last group consisted of perspective and review studies in the field (yellow slice), either methodologically or medically, which accounted for 21% of the selected articles. Figure 3B, in particular, illustrates the classification of XAI applications in neuroimaging in terms of the *post-hoc* method they used (along with their percentage). As mentioned in the literature, these methods can be divided into decomposition-based and perturbation-based approaches; the latter can be classified into gradients, signal and model agnostic ones.

Moreover, Figure 3C distinguishes applied XAI studies in neuroimaging from various aspects such as method, imaging modality, sample size, and their publication trend in recent years. In this plot, each circle represents a study whose color determines the type of imaging modality such as EEG, fMRI, MRI, CT, and other (PET, ultrasound, histopathological scans, blood film, electron cryotomography, Aβ plaques, tissue microarrays, etc.), and its size is logarithmically related to the number of people/scans/images used in that study. By focusing on each feature, compelling general information can be extracted from this figure. For example, it can be noted that gradient-based methods cover most imaging modalities well, or those model agnostic methods are suitable for studies with large sample sizes.

A summary of the reviewed articles is provided in Table 2, which contains the author's name, publication year, XAI model examined, and key contributions, respectively, ordered by taxonomy and article date. The table is constructed to provide the reader with a complete picture of the framework and nature of components contributing to XAI in medical applications.

**Table 2:** Summary of included articles by taxonomy, authors (year), XAI model, and key contributions.

**References**	**XAI model**	**Key contributions**
**XAI: Technical and theoretical concepts**		
Robnik-Šikonja and Kononenko (2008)	N/A	The paper proposes a method to explain predictions made by various ML models that output prediction probabilities separately for individual instances. The authors propose three types of explanations: the instance explanation, i.e., the explanation as to why a given instance is classified as it is, the model explanation, i.e., “averages of explanations over many training instances,” and the domain explanation, achievable “if the accuracy of the model is high.” The work also proposes a way to visualize the resulting explanations. However, the method is limited to a case in which only a single change in the variables is sufficient to change the prediction. The quality of the proposed explanations is also correlated with the quality of the model. An example of method performance is presented for a well-known Titanic dataset.
Erhan et al. (2009)	AM	The work elaborates on techniques to visualize higher-layer features learned by DL models. The authors recalls that the visualization of early layers (first, second) is commonly possible, especially in AI vision, by visualizing the filters learned by the DL models. They also propose an optimization technique called activation maximization (AM). Two other previously known methods, i.e., a sampling technique and a method that creates a linear combination of selected filters from previous layers, are also analyzed. The methods are tested on the MNIST dataset and a large collection of tiny 12 × 12 natural images. The results show that AM allows the visualization of features learned by deeper layers of the DL architecture and that these layers, in fact, learn more complex patterns than earlier layers of the DL model.
Gaonkar and Davatzikos (2013)	N/A	The paper proposes an “analytical short-cut” for the computation of explanations of SVM predictions. The method is demonstrated by analyzing fMRI data. In opposition to computation-expensive permutation tests, this method allows the data required to visualize 3D brain maps of statistically significant regions that contribute to the SVM prediction to be computed within a few seconds. The method is evaluated on simulated data, Alzheimer's disease data, and fMRI lie-detection data.
Simonyan et al. (2013)	AM, Gradient-based	This work addresses the visualization of CNNs trained for image classification by two techniques: AM and a method for computing the sample-wise saliency maps. The AM method is derived from literature, and the method for obtaining saliency maps is based on the computation of a single back-propagation pass. The results of both methods are visualized on randomly selected images from the ILSVRC-2013 test set. Demonstrates also that the created saliency maps can be used for “weakly supervised” object segmentation.
Zeiler and Fergus (2014)	DeConvNets	The paper proposes a new method for visualizing the function of intermediate feature layers in DL CNNs. The study profits from previous work, which introduced “deconvolutional networks.” This work compares the output from the proposed method to that of a simpler sensitivity analysis. The article presents transparent figures that help aid in the reader's understanding of the whole method. For various input images presents example outputs at different layers of the analyzed CNN. Another interesting result is the presentation of how learned features vary across layers and CNN training epochs.
Bach et al. (2015)	LRP	The paper introduces Layer-Wise Relevance Propagation (LRP), an algorithm that generates heatmap visualizations of the contribution of single pixels in predictions made by non-linear DL classifiers. Relevance scores are computed by the method and assigned to the analyzed pixels of the input image. The authors present numerous examples for various datasets (e.g., Pascal VOC2009, MNIST digits, ILSVRC) and classifiers. In addition, model and LRP performance when inferring based on images with flipped pixels are analyzed. The authors present how the choice of the hyperparameters alpha and beta influence the LRP output.
Ribeiro et al. (2016)	LIME	This work proposes a method “local interpretable model-agnostic explanations” (LIME) that enables the predictions “for any classifier in an interpretable and faithful manner, by learning an interpretable model locally around the prediction,” to be explained. LIME is demonstrated by explaining various models for text analysis and image classification. LIME is based on local simplifications of complex non-linear global solutions learned by the explained model. The authors also propose a variant of the method, called SP-LIME, that addresses the problem of “trusting the model” by selecting representative instances with explanations. The paper also names the desired characteristics for explainers, e.g., interpretability, local fidelity, and being model-agnostic.
Samek et al. (2016)	LRP	This paper summarizes LRP, originally published in another paper. This technique explains predictions carried out by DNN models in the image analysis domain.
Zhou et al. (2016)	CAM	This paper focuses on the advantages of the global average pooling (GAP) layer proposed for CNNs. The study shows that even when only given image-level labels, the CNN is capable of acquiring a remarkable localization ability during the learning process. The findings are proven and visualized by a class activation mapping (CAM) technique that enables instance-level visualization of features that contribute to the decision of the classifier. The authors argue that their visualization method is superior to previous methods, e.g., because it retains most of the CNN performance by only removing the fully-connected layers. In this work, CAM is compared to a similar approach based on global max pooling (GMP) and proves to be superior in localization. The performance of the method is demonstrated on various image datasets and classification tasks.
Arras et al. (2017a)	LRP	This paper applies LRP to explain the predictions carried out by CNN and bag-of-words-based SVM models in text classification tasks. In addition to utilizing the novel technique in a new domain, the authors utilize scores mapped based on words provided by the method to generate novel vector-based representations of texts. Based on these representations, a measure of “model explanatory power” is introduced, which shows for the two analyzed models, that CNN provided a slightly higher level of explainability.
Arras et al. (2017b)	LRP	This work extends the usage of the LRP method to RNN networks, e.g., LSTMs and GRUs. The method outputs text-heatmaps to visualize explanations. For example, a trained biLSTM text analysis model is used to carry out a five-class sentiment classification task, and the model's predictions are analyzed by proposed technique, as previously used in the domain sensitivity analysis (SA). The outputs of both methods are compared and discussed. In addition, quantitative analysis of extended LRP and SA is carried out by validation in a two-word deleting experiment in which the two most informative words have been deleted from the inferred text, and the trained model is asked to repeat its prediction.
Zintgraf et al. (2017)	PDA	The work identifies two types of methods for explaining DL image analysis predictions: AM and saliency methods. The authors introduce a saliency method called “prediction difference analysis.” For a given picture, the method highlights pixels that have played an important role for or against the prediction that the DL has made. The method relies on assessment of the probability of obtaining a correct prediction given a partially occluded input image and turns out to be time consuming, requiring a minimum of 20 min per image of computational time. It is possible to apply the method to selected layers of DL architecture. The authors present example results for natural images as well as brain MRI scans.
Montavon et al. (2017)	DTD	This paper introduces a saliency method for explaining the predictions of DL architectures, especially image classification, and is called “Deep Taylor Decomposition (DTD).” The method is compared to sensitivity analysis and is shown to provide more insight when applied to MNIST digits and ILSVRC images as well as CaffeNet and GoogleNet pre-trained models.
Kindermans et al. (2017)	GI, IG, DTD, SG, LRP	This work elaborates on the imperfections of the explanations of DL predictions provided by saliency methods. The paper quotes other research stating that before adopting an explanation, the method providing the explanation should be tested for completeness, implementation invariance, and sensitivity. “Input invariance” is proposed as another requirement. The authors carry out a comparison of various methods on MNIST images and find that many of these tested saliency methods do not maintain reasonable results after a simple shift in the input image. They also provide examples of deliberate misleading manipulation.
Sundararajan et al. (2017)	IG	The authors define axioms for explaining methods that must be satisfied as sensitivity and implementation invariance. Furthermore, the authors show that previous methods break either one axiom or the other. The authors also propose their own method, called Integrated Gradients, which aims to satisfy both axioms. The paper provides instructions for use and examples in the medical domain. The Integrated Gradients method is also shown to be effective in the NLP domain.
Lundberg and Lee (2017)	SHAP	This paper presents a unified framework for interpreting predictions from ML and DL algorithms that is called SHapley Additive exPlanations (SHAP). The authors define a new, general group of explainability models called additive feature attribution models and show that each of the six methods, namely LIME, DeepLIFT, LRP, Shapley regression values, Shapley sampling values, and Quantitative Input Influence (QII), fits the generalization. The work further elaborates on the computation of SHAP values and presents a comparison of feature importance as computed by the various methods.
Samek et al. (2017a)	SA, LRP	This article argues that the explanation of DL predictions should be a requirement, based on the following rationale: the need for verification, improvement, learning from the system, and compliance with legislation. Later, this paper compares heatmaps computed by SA and LRP for a DNN trained for image classification and presents LRP relevances per frame in human action recognition in videos.
Selvaraju et al. (2017)	Grad-CAM	The paper proposes a technique for creating visual explanations for inferences made by CNNs, called gradient-weighted class activation mapping (Grad-CAM). The paper identifies that “interpretability matters,” debates on “what makes a good explanation?” and discusses a trade-off between faithfulness and interpretability. The authors evaluate their method “via human studies.” Grad-CAM is a generalization of CAM that can be used with fully-convolutional layers, which are various networks that can be used not only for image classification but also for image captioning or visual question answering. The authors use guided backpropagation with Grad-CAM for fine-grained feature importance.
Shrikumar et al. (2017)	DeepLIFT	The article introduces a method called deep learning important features (DeepLIFT) that assigns importance scores to elements of input images thus explaining the predictions made by DNN. The authors divide similar methods into two groups, namely: (1) perturbation-based forward propagation approaches, and (2) backpropagation-based approaches, and categorize DeepLIFT into the second group, which is less expensive computationally. The paper further discusses critical previous backpropagation-based approaches. The proposed solution is based on the concept of difference-from-reference.
Doshi-Velez and Kim (2017)	N/A	This work focuses on the need to establish a consensus on what model interpretability is and to define an objective measure of interpretability. The authors divide current interpretability evaluations into two groups: (1) if the system is useful, then it must be interpretable, and (2) some model classes claim to be interpretable, i.e., rule list or sparse linear models. Later, the paper names the various desirable features of ML: fairness, unbiasedness, privacy, reliability, robustness, causality, usability, and trust. The authors argue that “the need for interpretability stems from an incompleteness in the problem formalization.” The paper also provides a taxonomy of interpretability evaluation and discusses open problems in the science of interpretability.
Smilkov et al. (2017)	SG, Gradients, IG, GBP	This paper introduces an improved version of gradient-based visual instance-level explanations for CNN predictions, called Smoothgrad (SG). The improvement visually sharpens the output of previous gradient-based methods. The method is based on the assumption that the ReLU activation functions commonly used in CNNs are usually not continuously differentiable and, as a result, proposes “smoothing” local discontinuities by computing “a simple stochastic approximation.” The method is compared to gradients, IG and Guided BackProp and is demonstrated to provide improved visualizations.
Adebayo et al. (2018)	Gradients, GI, IG, GBP, Guided Grad-CAM, SG	This article reflects critically on various gradient-based methods for generating saliency maps for image-based predictions. The authors define two concrete tests for the scope and quality of explanation methods (a model parameter randomization test and a data randomization test) and conduct extensive experiments with their use. According to the results of randomizing the weights in some layers of the tested CNN, some methods are independent both of the model and of the data and thus can provide misleading information. For example, Grad-CAM and Gradients passed the proposed tests, whereas Guided Grad-CAM failed.
Došilović et al. (2018)	N/A	This paper reviews the recent progress in XAI. The authors define notions that are important for this field: trust, interpretability, comprehensibility, explainability, and transparency and observe that some of these notions are nearly synonyms, while interpretability lacks a unique definition. This work recognizes two approaches to interpretability: integrated in the model structure and *post-hoc*. For the first group, a trade-off between model performance and “readability” is observed. In the second group, the authors distinguish methods that try to establish interpretability, predominantly, on a model-level, whereas another focuses on explainability at the instance-level. The utility of abstracted explanations for artificial general intelligence (AGI) is also discussed.
Hoffman et al. (2018)	N/A	This paper tries to explain questions regarding how the quality of XAI explanations can be measured. The paper depicts a conceptual model of the process of explaining AI that takes into consideration various XAI measurement categories: XAI goodness and user satisfaction, the user's mental model (the user's understanding of the AI system), curiosity (understood as XAI's ability to stimulate the user's curiosity, leading to improvement, for example, in the user's mental model), trust (the authors believe that trust in XAI will always be exploratory, i.e., will be based on allowing the user to explore the AI decision system), and performance. The authors indicate that the evaluation of the performance of an XAI system “cannot be neatly divorced from the evaluation of the performance of the user, or from the performance of the human-machine system as a whole.”
Montavon et al. (2018)	SA, AM, DTD, DeConvNets, GBP, LRP	The paper focuses on both the *post-hoc* interpretability of a pre-trained model, as opposed to incorporating interpretability in the model structure, and functional understanding instead of “mechanistic or algorithmic understanding.” The paper also analyses the model by explaining individual predictions. The authors create prototype data instances that will maximize activations (AM) of the analyzed DNN. The authors state that heatmap visualizations are more complete than images obtained through sensitivity analysis. The paper also describes good practices regarding DNN design if interpretability is to be achieved.
Ghorbani et al. (2019a)	ACE	This work proposes to go beyond per-sample-based explanations of ML predictions, discussing the principles and desiderata for concept-based explanations, before proposing a new algorithm for the automated concept-based explanation (ACE) of visual concepts. The ACE for an analyzed class analyzes many sample images, for each carrying out multi-resolution segmentation, clustering of similar segments, and computation of importance scores for each cluster by means of testing with the concept activation vectors (TCAV) method.
Lapuschkin et al. (2019)	LRP, SpRAy	This study presents how some example image classifiers achieve correct predictions through wrong features attributed to the dataset and not the objects themselves. The visualization of explanations is carried out with LRP. The work also uncovers a locally correct solution for AI trained to play a pinball game, which is not globally correct. Finally, the work proposes spectral relevance analysis (SpRAy), a semi-automated method for the analysis of AI behavior in large datasets.
Bosse et al. (2022)	CRP	Relevance Propagation (CRP) - A XAI method leveraging and extending the earlier LRP method and the concept of Activation Maximization to provide insights regarding both image-particular features contributing to the model's prediction as global features that the model learned to value and interpret. Owing to the design, the method allows one to quickly understand the general concept the model considers essential and the realization of that concept in the particular image in question.
**XAI in medical imaging applications**		
Mourão-Miranda et al. (2005)	Spatial maps	This work compares the performance of SVM and Fisher Linear Discriminant (FLD), in the assessment of brain states based on fMRI data without the prior selection of spatial features. Two functional tasks are analyzed, and PCA is employed for dimensionality reduction. During training, classifiers identify which voxels constitute “discriminating volumes,” i.e., features, that provide the most information needed for the classification of brain state. Provides visualizations of differences between classifiers in terms of the chosen discriminating volumes.
Kriegeskorte et al. (2006)	Searchlight	The paper introduces a method for the visualization of fMRI data, called “searchlight,” which provides an answer to the question “where in the brain the regional spatial activity pattern differs across experimental conditions.” Searchlight allows a continuous map in which informative regions are marked by moving a spherical multivariate “searchlight” through the measured volume to be obtained. This work demonstrates the utility of the method in regard to artificial fMRI signals and then further applies the method to real fMRI signals.
Wang et al. (2007)	Spatial maps	The authors analyze fMRI scans from multiple subjects by means of SVM and random effects analysis. The authors extract differences in brain activity between tasks in the form of a spatial discriminance map (SDM, also called “discriminating volume”) by means of SVM. To assess between-subject differences, the authors utilize both random effects analysis and permutation testing. The authors also propose group-level analysis, which is applied to a sensory-motor task with fMRI data.
Blankertz et al. (2011)	Spatial maps	This paper elaborates on the classification of human activity based on event-related potentials (ERP) in EEG data. This paper proposes a framework for signal preprocessing and highlights shrinkage estimators as a tool for improving later linear discriminant analysis (LDA). The improvements are presented in an evaluation experiment carried out on continuous EEG signals with the results compared to other models from the LDA family.
Haufe et al. (2014)	Spatio-spectral decomposition	Given the functional brain analysis domain and the struggle to model brain signals, this paper proposes a procedure for transforming “backward models” into “forward models” to enable the neurophysiological interpretation of the parameters of linear “backward models.” The considerations are valid for both EEG and fMRI data. The authors demonstrated on simulated and real fMRI and EEG data that the simple analysis of extraction filters may lead to severe misinterpretation in practice, whereas the proposed method for analyzing activation patterns resolves the problem.
Sturm et al. (2016)	LRP	This study proposes the application of DNNs with LRP for the first time for EEG data analysis. With the use of LRP, sample DNN decisions are transformed into relevance heatmaps. The authors found that DNN's performance is comparable to that of previously utilized approaches with CSP and LDA methods and that for low-performing subjects, transferring the learning of a DNN from another subject can improve the results.
Schirrmeister et al. (2017)	Correlation maps	This work studies the application of CNNs for decoding imagined or executed tasks from raw EEG data and compared the CNN's performance to a broadly used approach that utilizes a filter bank common spatial patterns (FBCSP) algorithm. The paper also benefits from visualization techniques that enable explanations regarding which features in the EEG data are informative for the CNN. A shallow and deep version of the CNN is designed for the study, in addition to a mixture of these two models, called “hybrid CNN,” and a ResNet-like CNN. Using an example dataset, the authors demonstrate that CNN's performance is similar to that of the FBCSP approach; however, applying recent improvements in the design and training of the CNNs allows this approach to achieve superior results.
Herent et al. (2018)	Correlations maps, occlusion-based heatmaps	This study analyses a very large sample (almost 1,600 patients) of MRI data to train brain age predictors. Various ML models are trained. Many preprocessing techniques are presented. In addition, raw data are used to fine-tune a CNN model. To explain the causes of predictions, the authors utilize tools like correlation maps, weights maps, and heatmaps with occlusion methods. A 2D CNN model provides the smallest error of 3.6 years of brain age.
Li et al. (2018)	Corrupting	The work focuses on the application of DL in the prediction of autism spectrum disorder (ASD) from fMRI data. The authors observe that recent studies have applied DL in this domain; however, such studies have also lacked model transparency. This paper proposes a simple CNN network and a time-window sliding technique to capture the time-spatial characteristics of the analyzed fMRI. The proposed approach is based on “corrupting” sections of the original images and assessing the change in the prediction of a model that has been previously trained on original non-corrupted data. The framework is first tested with synthetic data and then with real fMRI data.
Paschali et al. (2018)	Extreme cases	This work tests models created for carrying out predictions in the medical field by assessing inferences obtained from images with “extreme cases of noise, outliers and ambiguous input data.” The rationale for such specific testing is provided as “existing model evaluation routines look deeply into over-fitting but insufficiently into scenarios of model sensitivity to variations of the input.” The authors present several strategies for creating “adversarial examples” of images and state that even though the human eye can hardly catch the difference in a manipulated image from its original, nonetheless a pre-trained model can be easily fooled to change its prediction.
Yang et al. (2018)	SA-3DUCM, 3D-CAM, 3D-Grad-CAM	This paper proposes 3D extensions of methods for creating 2D visual explanations in CNN image analysis and applies these methods to the 3D CNN analysis of MRI scans from patients suffering from Alzheimer's disease. The authors propose three methods for explaining the black-box predictions of the utilized 3D CNNs: sensitivity analysis in 3D (SA-3DUCM), 3D class activation mapping (3D-CAM), and 3D gradient-weighted class activation mapping (3D-Grad-CAM). The authors observe that each explanation technique produces very different visualizations and conclude that improvement is still needed in the field of 3D DL explainability tools.
Zhao et al. (2018)	3D-Grad-CAM, Respond-CAM	This work proposes a novel method called Respond-CAM for creating explanations and their visualizations of predictions inferred by DL 3D image analysis models. The method is tested on 3D images of macromolecular complex structures obtained from Cellular Electron Cryo-Tomography (CECT). The authors argue that this dataset provides large variations in shapes and sizes, which is favorable for performance validation. Tests are conducted on two CNN models with the results compared with the Grad-CAM 3D visualization method. According to the results, the introduced Respond-CAM outperforms the 3D Grad-CAM visualization method.
Qin et al. (2018)	Attention maps	This article describes autofocus convolutional layer (ACL) for 3D CNNs to provide scale-invariance, especially in the biomedical domain of fMRI and CT data. The authors propose the ACLs can be added to existing 3D CNN models. The ACLs compute attention maps, which are utilized for creating the visualizations of features that are important in the model's decision process. As an example, a 3D CNN model is modified with ACLs to demonstrate the whole concept on the real fMRI and CT data.
Couture et al. (2018)	Cropping	This paper discusses Multiple Instance (MI) learning for breast tumor histology. The authors propose a method of interpretability that by cropping areas of selected size of the original image and learning the classifier based on them can output the importance of each single prediction to the final “bag” prediction. The final prediction is obtained through aggregating instance predictions by pooling with here proposed quantile function. The importance of image augmentation is also highlighted.
Thomas et al. (2019)	DeepLight, LRP	This work introduces DeepLight, a CNN feature extractor+LSTM-based DL framework for the analysis of fMRI scans. The scanned brain slices are aligned in a sequence processed by the LSTM. Visual explainability is maintained by the use of LRP. The method is tested on a large, 100-patient fMRI dataset. The method is compared to the baseline General Linear Model (GLM), Searchlight, and whole-brain lasso models. The authors underscore that the proposed LSTM method is an improvement over previous techniques due to its use of a data-driven identification of the time component in the spatial fMRI analysis instead of hand-crafted methods for the identification of time dependency.
Tang et al. (2019)	Guided Grad-CAM, feature occlusion	This paper proposes a proof-of-concept DL framework for the classification of images that are important for the diagnosis of Alzheimer's disease and the visualization of explanations of DL predictions. Initially, this study compares the framework of a human expert analysis of images related to predicting Alzheimer's disease with CNN models. The authors test many CNN architectures and hyperparameters and note that a relatively shallow CNN has achieved “strong classification performance.” The obtained results show impressive performance of the automated method. In addition, heatmap explanations generated by Guided Grad-CAM and feature occlusion are discussed.
Lee et al. (2019)	CAM	This paper proposes a DL framework with an ensemble of four pre-trained CNN models to address the task of predicting acute intracranial hemorrhage based on a dataset of 904 CT non-contrast head scans. The framework's original design allows it to mimic the human radiological workflow at the image preprocessing stage. The model showed a comparable performance to that of radiologists. This work provided activation maps generated by the CAM method, which were separately validated by radiologists. They also report an attempt to train a 3D model to carry out the same tasks; however, the authors report a very lower mean average precision (mAP) of the 3D model, attributing this fact to “the curse of dimensionality.”
Wang et al. (2019)	Activation patterns	This paper trains a CNN model to classify six hepatic tumor entities using 494 lesions on multi-phasic MRI. A *post-hoc* algorithm inferred the presence of imaging features in a test set of 60 lesions by analyzing activation patterns of the pre-trained CNN model and scoring of radiological features. The developed system accomplishes 76.5% positive predictive value in identifying the correct radiological features present in each test lesion for liver tumor diagnosis.
Palatnik de Sousa et al. (2019)	LIME	In this research LIME method is used to provide explanations to two CNN models deployed on the task of classification of lymph node metastases based on medical images from a publicly available data set.
Böhle et al. (2019)	LRP, Guided Backpropagation (GB)	The study uses LRP and GB XAI method to provide explanations of predictions carried out by a Deep Neural Network deployed on MRI data regarding Alzheimer's disease. The authors demonstrate that the LRP technique is superior to GB in the analyzed task.
Papanastasopoulos et al. (2020)	IG, SG	This paper applies XAI techniques, including IG and SG, to the regions-of-interest from the training set. They trained a CNN for the classification of estrogen receptor status (ER+ and ER–) to aid in the molecular classification of breast cancer based on MRI medical imaging. Their model lets the CNN select features from various complementary characteristics of the same patient images.
Essemlali et al. (2020)	Saliency maps	This work introduces a XAI experiment to better understand the connectomic structure of the Alzheimer's disease. They showed that deep learning over structural connectomes are a prevailing method to leverage connectomes in the complex structure derived from diffusion MRI tractography. The article understands the brain connectivity based on the different brain's alteration with dementia with saliency map extraction. The introduced procedure revealed that no single region is responsible for Alzheimer's disease, but the combined effect of several cortical regions.
Windisch et al. (2020)	Grad-CAM	This paper focuses on a neural network to differentiate between MRI slices containing either a vestibular schwannoma, a glioblastoma, or no tumor for a basic brain tumor detection. The Grad-CAM is implemented in their study to find the areas that the neural network based its predictions on. To assess the confidence of the model in its predictions, the Bayesian neural network approach is considered.
Meske and Bunde (2020)	LIME	The study discusses how XAI allows to improve the degree of AI transparency on the example of detecting malaria from medical images. A simple Multi-Layer Perceptron and CNN models are trained and LIME is used to demonstrate heatmaps on the original input images.
Nigri et al. (2020)	Swap Test	This research proposes a novel Swap Test technique to provide heatmaps that depict areas of the brain most indicative of the Alzheimer's disease based on predictions from CNN models carried out on MRI brain data. The new technique is compared to the occlusion test and by measures of continuity and selectivity is determined to be superior.
El-Sappagh et al. (2021)	SHAP	This article is developed a two-layer model with random forest (RF) as classifier algorithm that enhances the clinical understanding of Alzheimer's disease diagnosis and progression processes. The developed model provides physicians with accurate decisions along with a set of explanations for every decision. They implemented 22 explainers for each layer based on a decision tree classifier and a fuzzy rule-based system.
Lee et al., 2019	SLIC, LIME	This study introduces a DL classification system for breast cancer detection based on ultrasound images. The models were trained on a data set of images obtained from 153 patients. The proposed approach merges a known pixel segmentation method (named, SLIC) and LIME XAI technique and applied them to an ultrasound image already segmented by a trained DL segmentation model which allows LIME to highlight the meaningful fragment of the segmented image.
Binder et al. (2021)	LRP	This paper studied a XAI technique for the integrated profiling of morphological, molecular and clinical features from breast cancer histology. The LRP-heatmaps they compute diverge because attention computes a weight in the forward pass without considering the final prediction made further in the predictor. It facilitates the quantitative evaluation of histomorphological features, the prediction of multiple molecular markers for subsets of cases with high accuracy (>95%) and can relate morphological and molecular properties in terms of cancer biology.
Pennisi et al. (2021)	Grad-CAM, Var-Grad	This study proposes a novel deep learning approach to classification of COVID-19 based on CT scans. The experiments are verified with use of saliency maps generated by a mixture of two XAI methods.
Zhang et al. (2021)	3D Grad-CAM	A novel model is proposed in this study which is capable of predicting Alzheimer's disease based on 3D structure MRI data. The proposed approach allows end-to-end learning, automated diagnosis and provides 3D class activation mapping heat-maps. The method achieves superior results when compared to selected 3D Deep Neural Networks.
**Perspective and review studies**		
Holzinger et al. (2014)	N/A	This review gathers thoughts regarding knowledge discovery and interactive datamining in bioinformatics and elaborates on the enormous amounts of data available and the means to benefit from them. One thought is that given the abundance of data, there is insufficient bandwidth to focus on all of it. Another thought points out that the recent ML and AI algorithms lack interpretability, and as a result, should be treated with caution. Usability and interaction with the introduced methods are also named as important challenges. Describes four future areas of research in the domain: interactive data integration, data fusion and preselection of data-sets; interactive sampling, cleansing, preprocessing, mapping; interactive advanced datamining methods, pattern discovery; and interactive visualization, human-computer interaction, analytics, decision support.
Holzinger (2016)	N/A	This work elaborates on ML in the medical field, especially in interactive machine learning (iML), a group of algorithms that can interact with agents, possibly humans, for optimization of their learning process. The author argues that achieving a fully automatic ML is difficult in medical applications due to the fact that biomedical data-sets are full of uncertainty, incompleteness, and other flaws. The author states that a space for “human in-the-loop” methods is created, in which expert knowledge can help ML algorithms. The paper identifies the basis of iML as reinforcement learning (RL), preference learning (PL), and active learning (AL).
Biran and Cotton (2017)	N/A	This study reviews research concerning the explainability and justification of ML. The paper identifies the connected terms, interpretability, explainability, and justification and provides information regarding the historical approaches to the treatment of those terms in many ML-related fields. This review identifies two areas of work regarding explainability: the interpretation and justification of predictions and interpretable models. Model-specific and model-agnostic solutions are also identified. The authors observed that, especially in the NLP field, research has been focused on selecting a small part of the input as evidence to justify the predictions. The paper also identifies a model approximation, which “focuses on deriving a simple, interpretable model that approximates a more complex, uninterpretable one,” both in NLP and image classification.
Holzinger et al. (2017)	N/A	The authors dispute the need for research on XAI and name the European General Data Protection Regulation as one of the reasons. The authors also observe a trade-off between model performance and explainability. The authors further elaborate on explainability and other notions such as functional understanding, interpretation, and causality. Explainable models are divided into *post-hoc* and *ante-hoc*. The authors name Amplitude Modulation Frequency Modulation (AM-FM) decompositions as an example of creating explainable features in a medical image domain.
Lipton (2018)	N/A	The work discusses various notions regarding the interpretability of ML. The author underscores the fact that interpretability does not reference a monolithic concept. Importantly, the authors argue that especially in the medical field, “the short-term goal of building trust with doctors by developing transparent models might clash with the longer-term goal of improving health care.” The article also presents a warning regarding blindly trusting *post-hoc* interpretations, because they can potentially be misleading. The paper motivates researchers to clearly define notions regarding general interpretability each time they publish results and claim to achieve it.
Holzinger (2018)	N/A	This study is an overview of development from ML to XAI with an emphasis on the importance of human-computer interaction. The author introduces terms such as Automatic ML (aML), interactive ML (iML), and Human-Computer Interaction and Knowledge Discovery/Data Mining (HCI-KDD) and elaborates on several important elements of HCI-KDD: (1) data preprocessing and integration, (2) learning algorithms, (3) data visualization, (4) issues of data protection safety and security, (5) graph-based data mining, (6) topology-based data mining, and (7) entropy-based data mining. The author also mentions the struggle to achieve XAI.
Hosny et al. (2018)	N/A	This opinion article aims to familiarize a general understanding of AI methods, especially in regard to image-based tasks in radiology and oncology. The authors elaborate on AI capabilities in detection, characterization (a medical term referring to “the segmentation, diagnosis and staging of a disease”), monitoring, and other opportunities, e.g., data preprocessing or integrated diagnostics. The requirements regarding managing medical data are also discussed (Health Insurance Portability and Accountability Act - HIPAA), and positive examples are named.
Tjoa and Guan (2020)	N/A	This paper is a review of a non-exhaustive list of works regarding XAI in general and, specifically, in the medical domain. The authors find that many works that propose interpretability methods assume that such method provide obvious results that do not require human testing, which is believed to not always be true. The paper introduces various types of interpretability in AI and lists a few risks of their application in the medical domain.
Holzinger et al. (2019)	N/A	This work elaborates on the importance in the medical field of the ability to explain the elements that cause AI to output a given prediction. Differences between explainablity and causability are discussed. The paper distinguishes *post-hoc* and *ante-hoc* systems that enable the explanation of versions parts of the AI decision process. LIME is given as an example of the *post-hoc* system, whereas *ante-hoc* systems are interpretable based on their design, e.g., decision trees or fuzzy inference systems. The authors mention tools that are useful when interpreting DL predictions: uncertainty, attribution, activation maximization. The paper also presents an example of *post-hoc* and *ante-hoc* explanations by a human expert in a histopathological use-case.
Lundervold and Lundervold (2019)	N/A	This article is a survey of ML and DL applications in the medical domain, with a particular emphasis on the MRI field. The authors name numerous areas of AI application in medicine and observe that DL allows for a significant increase of the speed of computations and improvements in computational quality. It is noted that DL can be used for both diagnosis and signal processing. The authors also list medical imaging datasets in Arxiv and Github as the newest-information hubs, as well as various medical imaging competitions.
Langlotz et al. (2019)	N/A	This work identifies future key research areas in the AI Medical domain. The paper recalls the achievement of super-human performance by AI models in the classification of objects in 2015 during the ImageNet Large-Scale Visual Recognition Challenge. In addition, research opportunities in AI for medical imaging related to data sharing and data availability are discussed. Among various other topics, the need for “machine learning methods that can explain the advice they provide to human users” is highlighted.
Xu et al. (2019)	Various	This shallow review introduces the history of XAI, starting from expert systems and ML and progressing to the latest progress in DL-related XAI. The article gives an example of a DARPA-funded program for the development of XAI and depicts elements of current state-of-the-art and desiderata for future development, with a focus on the image analysis domain. This work also recalls the errors in trained models, as discovered by XAI. The paper ends with a discussion on the challenges and future directions.
Kohoutová et al. (2020)	N/A	This paper elaborates on interpreting ML models in neuroimaging. The authors classify ways to interpret these complex brain models, they should (i) be understandable to humans, (ii) deliver valuable information about what mental or behavioral constructs are represented in particular brain regions, and (iii) establish that they are based on the relevant neurobiological signal, not artifacts or confounds. The provided protocol will support more interpretable neuroimaging models, also the users should be familiar with basic programming in MATLAB or Python.
Singh et al. (2020)	N/A	This study contributes a common framework for comparison of 13 XAI attribution methods used for Ophthalmic Disease Classification based on medical imaging. It presents a thorough comparison of both quantitative and qualitative performance of the methods.
Lucieri et al. (2021)	N/A	This study reviews XAI methods and studies applied to the dermatology domain. It identifies four main groups of explanation approaches as Visual Relevance Localization, Dermoscopic Feature Prediction and Localization, Similarity Retrieval and Intervention.
Hryniewska et al. (2021)	N/A	This research carries out a systematic review of numerous studies predicting COVID-19 from medical images based on deep learning architectures which utilize XAI techniques and focuses on mistakes made at different stages of model development. Among other, the study highlights example errors in XAI explanations as observer by trained radiologist.
Joshi et al. (2021)	N/A	This paper describes and reviews the present literature to present a comprehensive survey and commentary on the different explainability methods and techniques in a multimodal deep neural net especially image and text modalities in vision and language settings. The paper covers numerous topics on multimodal AI and its applications for generic domains including the significance, datasets, fundamental building blocks of the methods and techniques, challenges, applications, and future trends in this domain.

N/A, not applicable.

### Co-occurrence analysis

**Figure 4:**
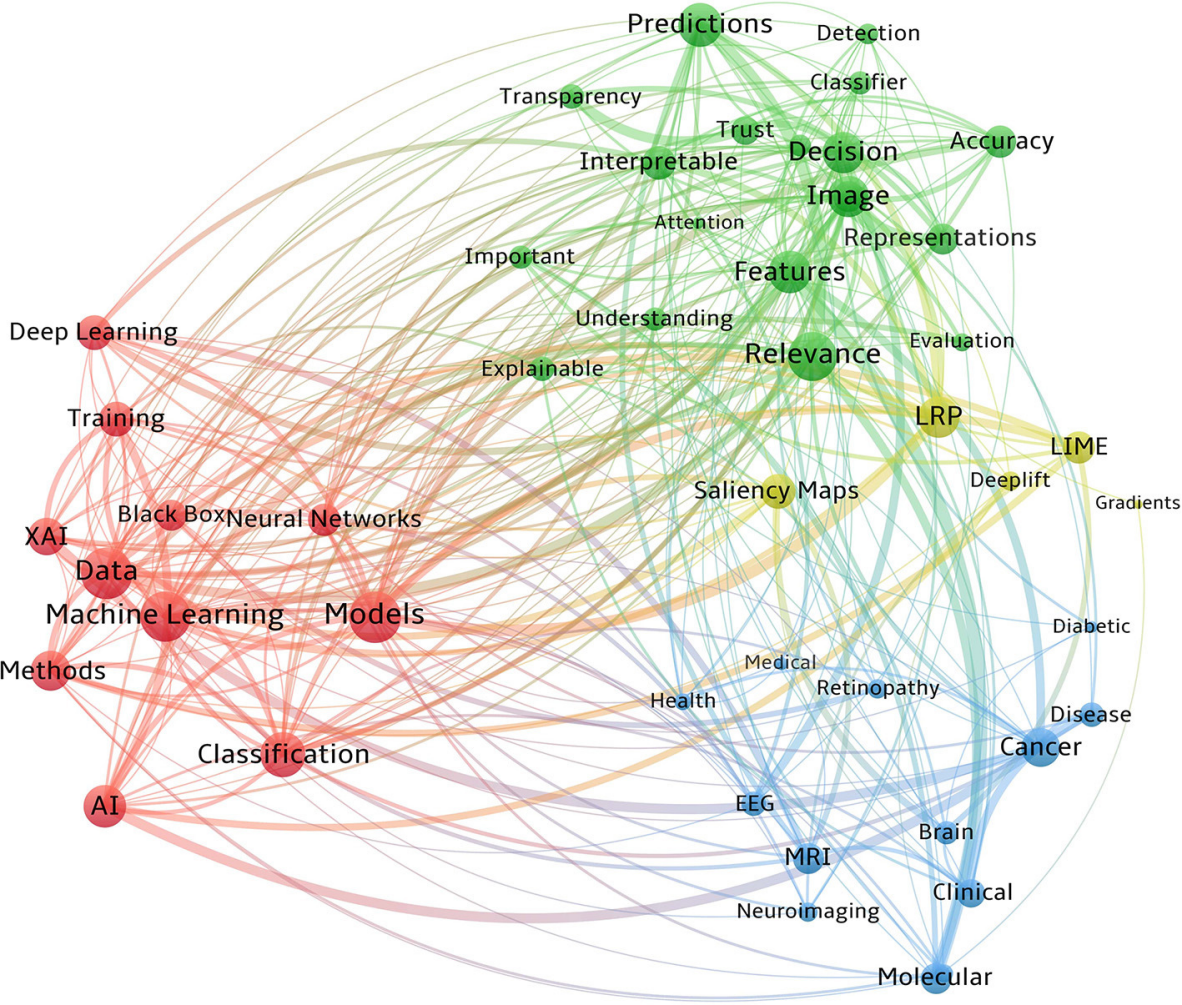
Co-occurrence network of the commonly used words in reviewed studies.

Counting of matched data within a collection unit is what co-occurrence analysis is all about. Figure 4 visualizes the co-occurrences of the key vocabularies of XAI/AI concepts, XAI methodologies, imaging modalities, and diseases from our reviewed papers. In this figure, word clusters are represented by different colors. Also, the bubble size denotes the number of publications, while the connection width reflects the frequency of co-occurrence. We only considered the abstracts in our co-occurrence analysis due to the large number of words within the full texts and the curse of overlapping labels.

### Reliability analysis: XAI tests/measures

XAI methods are likely be unreliable against some factors that do not affect the model outcome. The output of the XAI methods, for instance, could be significantly altered by a slight transformation in the input data, even though the model remains robust to these changes (Kindermans et al., 2019). Accordingly, we conducted a subjective examination on articles that have introduced properties such as completeness, implementation invariance, input invariance, and sensitivity (Bach et al., 2015; Kindermans et al., 2017; Sundararajan et al., 2017; Alvarez-Melis and Jaakkola, 2018) to evaluate the reliability of these methods (Table 3).

**Table 3:** XAI tests and measures.

**XAI methods**	**Test/measure**	**Outcome**	**References**
GI, IG, LRP	Ground-truth	LRP was shown to perform best, followed by IG.	Osman et al., 2020
LRP, LIME	Relevance structural similarity	Superiority of decomposition-based algorithms such as LRP over the others	Douglas and Farahani, 2020
LIME, SHAP	Robustness/Lipschitz Estimate	Both methods were robust for SVM, unstable for NN and RF; LIME was more unstable than SHAP	Alvarez-Melis and Jaakkola, 2018
Saliency maps, GI, IG, LRP, Occlusion Sensitivity, LIME	Robustness/Lipschitz Estimate	IG performed best, LIME very bad, rest satisfactory	Alvarez-Melis and Jaakkola, 2018
Gradients, GI, SG, DeConvNets, GBP, DTD, IG	Input invariance	DTD and IG pass “contingent on the choice of reference and the type of transformation considered”	Kindermans et al., 2017
DeepLift, LRP, IG	Implementation invariance	DeepLift and LRP fail, IG pass	Sundararajan et al., 2017
Gradients, DeConvNets, GBP, DeepLift, LRP, IG	Sensitivity	Gradients, DeConvNets, GBP fail, DeepLift, LRP, IG pass	Sundararajan et al., 2017
Gradients, GI, IG, GBP, Guided Grac-CAM, SG	Model parameter randomization test	Gradients and Grad-CAM pass, rest fail	Adebayo et al., 2018
Gradients, GI, IG, GBP, Guided Grac-CAM, SG	Data randomization test	GI, IG pass, rest fail	Adebayo et al., 2018
Gradients, IG, DeepLIFT	Fragility/adversarial input samples	Gradients and DeepLIFT are more fragile	Ghorbani et al., 2019a

### Quality assessment

**Figure 5:**
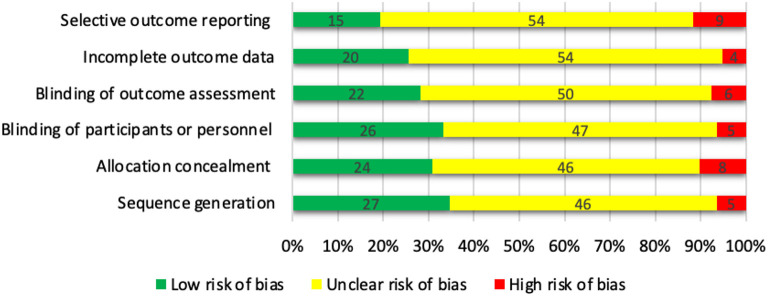
Assessing the risk of bias using the Cochrane Collaboration's tool.

The Cochrane Collaboration's tool (Higgins et al., 2011) was used to assess the risk of bias in each trial (Figure 5). The articles were categorized as: (a) low risk of bias, (b) high risk of bias, or (c) unclear risk of bias for each domain. We judged most domains to be unclear or not reported using the Cochrane Collaboration. Finally, the overall quality of the studies was classified into weak, fair, or good, if < 3, 3, or ≥4 domains were rated as low risk, respectively. Among the 78 studies included in the systematic review, 22 were categorized as good quality, 50 were fair quality, and 6 were low quality.

## Discussion

The current study provides an overview of applications of *post-hoc* XAI techniques in neuroimaging analysis. We focused on *post-hoc* approaches since interpreting weight vectors has historically been the standard practice when applying encoding and decoding models to functional imaging and neuroimaging data. However, it is generally challenging to interpret decoding and encoding models (Kriegeskorte and Douglas, 2019). In light of this, *post-hoc* procedures provide a novel strategy that would make it possible to use these techniques for predictions and gain scientific and/or neuroscientific knowledge during the interpretation step.

For many years, ML and DL algorithms have established a strong presence in various medical imaging research with examples of performance at least equaling that of radiologists (Khan et al., 2001; Hosny et al., 2018; Li et al., 2018; Lee et al., 2019; Lundervold and Lundervold, 2019; Tang et al., 2019). In contrast to linear models, many practitioners regard DNNs as a “black box,” and this lack of transparency has hindered the adoption of deep learning methods in certain domains where explanations are crucial (Guidotti et al., 2018b). Transparency builds trust, subtends the evaluation of fairness, and helps identify points of model failure (Kindermans et al., 2017; Rajkomar et al., 2018; Vayena et al., 2018; Wilson et al., 2019). In many cases, trustworthy models may be essential to verify that the model is not exploiting artifacts in the data, or operating on spurious attributes that covary with meaningful support features (Leek et al., 2010; Lapuschkin et al., 2016; Montavon et al., 2018).

The need for interpreting the black-box decisions of DNNs (Holzinger, 2014; Biran and Cotton, 2017; Doshi-Velez and Kim, 2017; Lake et al., 2017; Lipton, 2018) was answered by leveraging a variety of *post-hoc* explanation techniques in recent years. These models can assign relevance to inputs for the predictions carried out by trained deep learning models either for each instance separately (Robnik-Šikonja and Kononenko, 2008; Zeiler and Fergus, 2014; Ribeiro et al., 2016; Sundararajan et al., 2017) or on the class or model level (Datta et al., 2016; Guidotti et al., 2018a; Staniak and Biecek, 2018; Ghorbani et al., 2019b). Because of the successful applications of CNNs in image analysis, particularly in the medical domain, several XAI methods were proposed solely for explaining predictions of 2D (Springenberg et al., 2014; Bach et al., 2015; Smilkov et al., 2017) and 3D images (Yang et al., 2018; Zhao et al., 2018; Thomas et al., 2019).

This systematic review also reveals the need to involve medical personnel in developing ML, DL, and XAI for the medical domains. Without feedback from clinicians' active participation, it will be unlikely to create ML models dedicated solely to the medical fields (Ustun and Rudin, 2016; Lamy et al., 2019). Familiarizing AI researchers with the original needs and point-of-view of specialists from the medical domain and its subdomains (Tonekaboni et al., 2019) would be beneficial because it would allow focusing on the detailed shortcomings of the state-of-the-art XAI methods, followed by their significant improvement.

### Why is XAI needed in neuroimaging?

**Figure 6:**
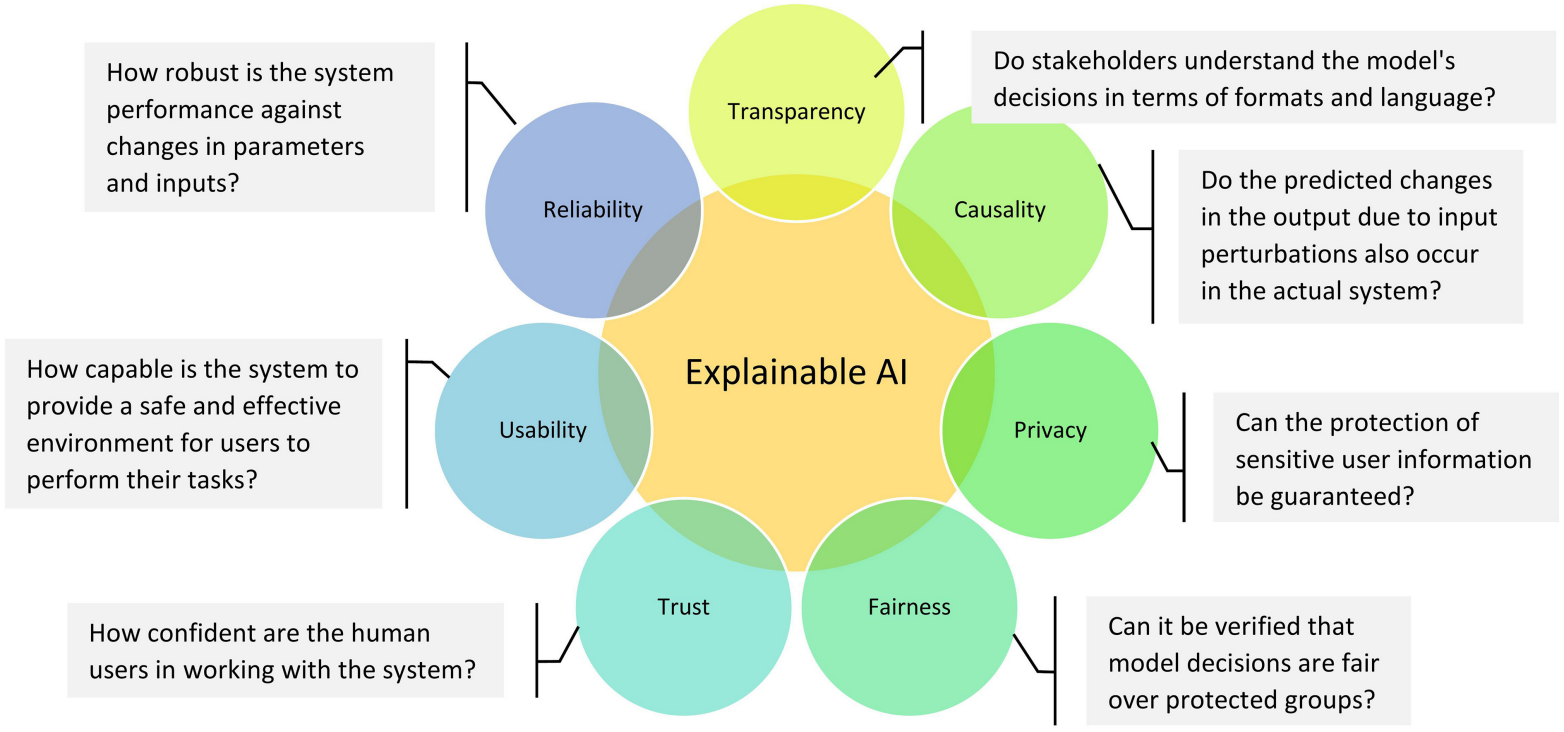
Requirement for interpretability in medical intelligent systems.

From the perspective of health stakeholders (e.g., patients, physicians, pharmaceutical firms and government), interpretability is an integral part of choosing the optimal model. As shown in Figure 6, interpretability could also be used to ensure other significant desiderata of medical intelligent systems such as transparency, causality, privacy, fairness, trust, usability, and reliability (Doshi-Velez and Kim, 2017). In this sense, *transparency* indicates how a model reached a given result; *causality* examines the relationships between model variables; *privacy* assesses the possibility of original training data leaking out of the system; *fairness* shows whether there is bias aversion in a learning model; *trust* indicates how assured a model is in the face of trouble; *usability* is an indicator of how efficient the interaction between the user and the system is; and *reliability* is about the stability of the outcomes under similar settings (Doshi-Velez and Kim, 2017; Miller, 2019; Barredo Arrieta et al., 2020; Jiménez-Luna et al., 2020; Fiok et al., 2022).

Even though AI methods have successfully been utilized in medical research and neuroimaging studies, these methods still have not advanced into everyday real-life applications. Researchers name several reasons for this fact: (1) Lack of interpretability, transparency, trust, and clear causality of the black-box AI models continues to be a vital issue (Holzinger et al., 2017; Došilović et al., 2018; Hoffman et al., 2018) despite the research already carried out on XAI. (2) Speeding up model convergence while maintaining predictive performance is important in scenarios where data is naturally homogeneous or spatially normalized (e.g., fMRI, MRI sequences, PET, CT). This is crucial for neuroimaging research since the data are relatively homogeneous, unlike natural images, because of their uniform structure and spatial normalization (Eitel et al., 2021). (3) Despite improvements in medical data availability for AI training (Hosny et al., 2018; Lundervold and Lundervold, 2019), an insufficient amount/quality of data for training ML and DL solutions remains a significant limitation, with the result that many studies are carried out on small sample sizes of subjects (13 in Blankertz et al., 2011; 10 in Sturm et al., 2016; 10 in Tonekaboni et al., 2019). (4) It is believed that trained AI models that achieve super-human performance on data from a distribution (e.g., a specific hospital) cannot adapt appropriately to unseen data drawn from other medical units since it comes from a different distribution (Yasaka and Abe, 2018). (5) Compliance with legislation that calls for the “right for explanation” is also considered (Holzinger et al., 2017; Samek et al., 2017b) to be a limiting factor regarding the use of ML and DL without the ability to provide explanations for each use case.

Understanding the need for XAI in the DNNs community seems to spread rapidly or can be already considered widespread. However, the importance of XAI, particularly for the medical domain, is still underestimated. When human health and life is at stake, it is insufficient to decide based solely on a “black box” prediction even when obtained from a superhuman model. It is far not enough to classify; instead, interpretation is the key to achieving if XAI manages to deliver a complete and exhaustive description of voxels that constitute a part of a tumor. The potential for XAI in medicine is exceptional as it can answer why we should believe that the diagnosis is correct.

### Evaluation of explanation methods

In recent years, various computational techniques have been proposed (Table 3) to objectively evaluate explainers based on accuracy, fidelity, consistency, stability and completeness (Robnik-Šikonja and Kononenko, 2008; Sundararajan et al., 2017; Molnar, 2020; Mohseni et al., 2021). *Accuracy* and *fidelity* (sensitivity or correctness in the literature) are closely related; the former refers to how well an explainer predicts unseen data, and the latter indicates how well an explainer detect relevant components of the input that the black box model operates upon (notably, in case of high model accuracy and high explainer fidelity, the explainer also has high accuracy). *Consistency* refers to the explainer's ability to capture the common components under different trained models on the same task with similar predictions. However, high consistency is not desirable when models' architectures are not functionally equivalent, but their decisions are the same (due to the “Rashomon effect”). While consistency compares explanations between models, *stability* compares explanations under various transformations or adversaries to a fixed model's input. Stability examines how slight variations in the input affect the explanation (assuming the model predictions are the same for both the original and transformed inputs). Eventually, *completeness* reveals a complete picture of features essential for decisions, so how well humans understand the explanations. It looks like the elephant in the room, that somewhat abstruse to measure, but very important to get right in future research. Particularly in medicine, we need a holistic picture of the disease, such as a complete and exhaustive description of voxels that are part of a tumor. What if altered medial temporal lobe shape covaries with a brain tumor (because the tumor moves it somehow)? Should we then resect the temporal lobe? Thus, further research is needed on this property.

In *post-hoc* explanation, fidelity has been studied more than accuracy (in fact, high accuracy is solely important when an explanation is used for predictions). In this respect, Bach et al. (2015) and Samek et al. (2017a) suggested a framework to evaluate saliency explanation techniques by pixel-flipping in an image input repeatedly (based on their relevance importance), then quantifying the effect of this perturbation on the classifier prediction. Their framework inspired many other studies (Ancona et al., 2017; Lundberg and Lee, 2017; Sundararajan et al., 2017; Chen et al., 2018; Morcos et al., 2018). The common denominator of fidelity metrics is that the greater the change in prediction performance, the more accurate the relevance. However, this approach may lead to unreliable predictions when the model receives out-of-distribution input images (Osman et al., 2020). To solve this problem, Osman et al. (2020) developed a synthetic dataset with explanation ground truth masks and two relevance accuracy measures for evaluating explanations. Their approach provides an unbiased and transparent comparison of XAI techniques, and it uses data with a similar distribution to those during model training.

Another possible way for appraising explanations is to leverage the saliency maps for object detection, e.g., by setting a threshold on the relevance and then calculating the Jaccard index (also known as Intersection over Union) concerning bounding box annotations as a measure of relevance accuracy (Simonyan et al., 2013; Zhang et al., 2018). However, since the classifier's decision is based solely on the object and not the background (contradictory to the real world) in this approach, the evaluation could be misleading. In many other cases, comparing a new explainer with those state-of-the-art techniques is utilized to measure explanation quality (Lundberg and Lee, 2017; Ross et al., 2017; Shrikumar et al., 2017; Chu et al., 2018).

On the other hand, Kindermans et al. (2019) proposed an *input invariance* property. They revealed that explainers might have instabilities in their results after slight image transformations, and consequently, their saliency maps could be misleading and unreliable. They assessed the quality of interpretation methods such as Gradients, GI, SG, DeConvNets, GBP, Taylor decomposition, and IG. Only Taylor decomposition and IG passed this property, subject to the choice of reference and type of transformation. In another study, Sundararajan et al. (2017) introduced two measures for evaluating the reliability of XAI methods, one called *sensitivity* (or fidelity) and the other as *implementation invariance* (i.e., a requirement that models with different architectures that achieve the same results should also provide the same explanations). In their paper, the sensitivity test was failed by Gradients, DeConvNets, and GBP, while DeepLift, LRP, and IG passed the test; in contrast, the implementation invariance was failed by DeepLift and LRP, while IG passed. To extend a similar idea, Adebayo et al. (2018) proposed another evaluation approach (to test Gradients, GI, IG, GBP, Guided Grad-CAM, and SG methods) by applying *randomizations tests* on the model parameters and input data, to confirm that the explanation relies on both these factors. Here, Gradients and Grad-CAM methods succeeded in the former and GI and IG in the latter. While these assessments can serve as a first sanity check for explanations, they cannot directly evaluate the explanation's adequacy.

One more known approach for evaluating visual explanations is to expose the input data to adversaries, unintentional or malicious purposes, which are generally unrecognizable to the human eyes (Paschali et al., 2018; Douglas and Farahani, 2020). For example, Douglas and Farahani (2020) developed a structural similarity analysis and compared the reliability of explanation techniques by adding small amounts of Rician noise to the structural MRI data (in the real world, this kind of adversary can be caused by the physical and temporal variability across instrumentation). In this study, while not significantly changing CNN's prediction performance for both the original and attacked images, the obtained relevance heatmaps showed the superiority of decomposition-based algorithms such as LRP over the others. In another study, Alvarez-Melis and Jaakkola (2018) proposed a Lipschitz estimate to evaluate explainers' stability by adding Gaussian noise to the input data. The authors showed SHAP was more stable than LIME when a random forest was considered. They also assessed explanations provided by LIME, IG, GI, Occlusion sensitivity, Saliency Maps, and LRP over CNNs. They reported acceptable results by all methods (IG was the most stable) but LIME. Finally, Ghorbani et al. (2019a) proposed to measure fragility, i.e., given an adversarial input image (perturbed original), the degree of behavioral change of the XAI method. In their work, Gradients and DeepLIFT were found to be more fragile than IG.

While there is an ongoing discussion regarding the virtues that XAI should exhibit, so far, no consensus has been reached, even regarding fundamental notions such as interpretability (Lipton, 2018). Terms such as completeness, trust, causality, explainability, robustness, fairness, and many others are actively brought up and discussed by different authors (Biran and Cotton, 2017; Doshi-Velez and Kim, 2017; Lake et al., 2017), as researchers now struggle to achieve common definitions of most important XAI nomenclature (Doshi-Velez and Kim, 2017). Given the research community's activity in this field, it is very likely that additional requirements and test proposals will be formulated shortly. Moreover, new XAI methods will undoubtedly emerge. We also note that not a single XAI method passed all proposed tests, and not all tests were conducted with all available algorithms. The abovementioned reasons force us to infer that the currently available XAI methods (Holzinger et al., 2022a,b), exhibit significant potential, although they remain immature. Therefore, we agree with Lipton (2018), which clearly warns about blindly trusting XAI interpretations because they can potentially be misleading.

## Conclusion

AI has already inevitably changed medical research perspectives, but without explaining the rationale for undertaking decisions, it could not provide a high level of trust required in medical applications. With current developments of XAI techniques, this is about to change. Research on fighting cardiovascular disease (Weng et al., 2017), hypoxemia during surgery (Lundberg and Lee, 2017), Alzheimer's disease (Tang et al., 2019), breast cancer (Lamy et al., 2019), acute intracranial hemorrhage (Lee et al., 2019) and coronavirus disease (Wang et al., 2019), can serve as examples of developing successful AI+XAI systems that managed to adequately explain their decisions and pave the way to many other medical applications, notably neuroimaging studies. However, the XAI in this research field is still immature and young. If we expect to overcome XAI's current imperfections, great effort is still needed to foster XAI research. Finally, medical AI and XAI's needs cannot be achieved without keeping medical practitioners in the loop.

## Data availability statement

The original contributions presented in the study are included in the article/supplementary material, further inquiries can be directed to the corresponding author/s.

## Author contributions

FF and KF conducted the literature search and prepared the initial draft of the paper. FF, KF, BL, and PD were involved in study conception and contributed to intellectual content. WK and PD supervised all aspects of manuscript preparations, revisions, editing, and final intellectual content. FF, KF, and BL edited the final draft of the paper. All authors contributed to the article and approved the submitted version.

## Conflict of interest

The authors declare that the research was conducted in the absence of any commercial or financial relationships that could be construed as a potential conflict of interest.

## Publisher's note

All claims expressed in this article are solely those of the authors and do not necessarily represent those of their affiliated organizations, or those of the publisher, the editors and the reviewers. Any product that may be evaluated in this article, or claim that may be made by its manufacturer, is not guaranteed or endorsed by the publisher.
